# Shock metamorphism in plagioclase and selective amorphization

**DOI:** 10.1111/maps.13494

**Published:** 2020-05-30

**Authors:** Lidia Pittarello, Luke Daly, Annemarie E. PickersgilL, Ludovic Ferrière, Martin R. Lee

**Affiliations:** ^1^ Department of Mineralogy and Petrography Natural History Museum Burgring 7 A‐1010 Vienna Austria; ^2^ Department of Lithospheric Research University of Vienna Althanstrasse 14 A‐1090 Vienna Austria; ^3^ School of Geographical and Earth Sciences University of Glasgow Gregory Building Lilybank Gardens Glasgow G12 8QQ UK; ^4^ Space Science and Technology Centre School of Earth and Planetary Sciences Curtin University GPO Box U 1987 Perth Western Australia 6845 Australia; ^5^ Australian Centre for Microscopy and Microanalysis University of Sydney Sydney 2006 New South Wales Australia

**Keywords:** amorphization, electron backscatter diffraction, deformation localization, plagioclase feldspar, planar deformation features, shock metamorphism

## Abstract

Plagioclase feldspar is one of the most common rock‐forming minerals on the surfaces of the Earth and other terrestrial planetary bodies, where it has been exposed to the ubiquitous process of hypervelocity impact. However, the response of plagioclase to shock metamorphism remains poorly understood. In particular, constraining the initiation and progression of shock‐induced amorphization in plagioclase (i.e., conversion to diaplectic glass) would improve our knowledge of how shock progressively deforms plagioclase. In turn, this information would enable plagioclase to be used to evaluate the shock stage of meteorites and terrestrial impactites, whenever they lack traditionally used shock indicator minerals, such as olivine and quartz. Here, we report on an electron backscatter diffraction (EBSD) study of shocked plagioclase grains in a metagranite shatter cone from the central uplift of the Manicouagan impact structure, Canada. Our study suggests that, in plagioclase, shock amorphization is initially localized either within pre‐existing twins or along lamellae, with similar characteristics to planar deformation features (PDFs) but that resemble twins in their periodicity. These lamellae likely represent specific crystallographic planes that undergo preferential structural failure under shock conditions. The orientation of preexisting twin sets that are preferentially amorphized and that of amorphous lamellae is likely favorable with respect to scattering of the local shock wave and corresponds to the “weakest” orientation for a specific shock pressure value. This observation supports a universal formation mechanism for PDFs in silicate minerals.

## INTRODUCTION

Plagioclase feldspar is the most abundant rock‐forming mineral in the crust of solid planetary bodies, including Earth, and represents up to 70% of the Moon’s crust (e.g., Papike et al. 1991). Despite the abundance of plagioclase, studies of its response to shock metamorphism are limited in comparison to those of quartz or olivine (e.g., Langenhorst [2002] and references therein). However, there has been a renewed interest in the study of shock effects in plagioclase, facilitated by improvements to analytical methods (e.g., Jaret et al. 2014, 2018; Fritz et al. 2019; Sims et al. 2019, 2020). The disparity in the number of studies is likely due to the relatively complex crystal structure of plagioclase in comparison to quartz, its variable response to shock depending on the amount of Ca versus Na in its structure (e.g., Stöffler et al. [2018], and references therein), and its susceptibility to secondary alteration, potentially obliterating shock features. For pressures in excess of the Hugoniot Elastic Limit for plagioclase (3.5–4.5 GPa), the following shock features have been observed as pressure increases (i) fracturing and mosaicism, (ii) formation of planar deformation features (PDFs) that manifest as thin (<1 μm thick) lamellae of isotropic material aligned along rational crystallographic planes, (iii) isotropization (i.e., transformation into diaplectic glass), and (iv) quenched melt (e.g., Huffman and Reimold 1996). These features correspond to a progressive breakdown of the plagioclase crystal lattice and have been recognized by the variable optical properties they generate in plagioclase (e.g., Stöffler 1967). Pre‐shock temperature of the impacted material controls to some extent the shock pressure necessary for these transformations to occur (Huffman and Reimold 1996). Shock‐induced planar microstructures, such as planar fractures (PFs) and PDFs, were first described and defined in quartz (e.g., Engelhardt and Bertsch 1969; Stöffler and Langenhorst 1994; Grieve et al. 1996; Langenhorst 2002). According to the definition proposed by these authors, PFs are open fractures, similar to cleavage planes, spaced >5 to 20 μm apart, but which form along rational crystallographic planes that are only activated by dynamic shock. By contrast, PDFs are thin (<200–300 nm) and closely spaced (<10 μm), originally amorphous lamellae, which have the same chemical composition as the host, developed along rational crystallographic planes, and unrelated to cleavage. The formation mechanism of PDFs in quartz is still not fully understood. Transmission electron microscopy has revealed that the optically isotropic (apparently amorphous) material in quartz PDFs can be either fully glassy or crystalline, and in the case of crystalline material, it locally contains micro‐twins or domains with a high density of dislocations (e.g., Goltrant et al. 1991). Diaplectic glass is commonly defined as the product of isotropization of a formerly crystalline phase, preserving the crystal’s shape but not its internal crystal structure. Isotropic plagioclase is commonly termed maskelynite (Tschermak 1872; Binns 1967). However, a debate exists about both the use of this term (e.g., Ferrière and Brandstätter 2015) and the nature of maskelynite itself, namely whether maskelynite is derived from a dense impact melt (Chen and El Goresy 2000), or formed through solid‐state collapse of the crystal structure (i.e., a “true” diaplectic glass; e.g., Fritz et al. 2005; Jaret et al. 2015). It is possible that in early works, the term maskelynite was used to define apparently amorphous plagioclase that may actually be a fine‐grained aggregate of hollandite, a high‐pressure polymorph of anorthite (Gillet et al. 2000).

The whole range of plagioclase compositions, which are expressed as molar % anorthite, abbreviated An, is represented in planetary material, from albite (An_0‐10_) and oligoclase (An_10‐30_) in L‐type ordinary chondrite meteorites, up to bytownite (An_70‐90_) and anorthite (An_90‐100_) in lunar rocks (e.g., Papike et al. 1991). The shock pressure required for the transformation of crystalline plagioclase into amorphous material is strongly dependent on the An content, with anorthitic compositions being amorphized at lower shock pressures than albitic compositions (e.g., Fritz et al. 2011; Stöffler et al. [2018] and references therein). Likewise, it seems that the occurrence of PDFs in plagioclase is also related to An content. For example, PDFs have been reported only for a limited range of compositions: including albite (in impactites from the El’gygytgyn impact structure, Russia; Pittarello et al. 2013), oligoclase (in the shocked L6 Tenham meteorite; Langenhorst et al. [1995], and in impactites from the Tenoumer impact structure, Mauritania; Jaret et al. 2014), doubtful features in andesine (An_30‐50_) in experimentally shocked plagioclase (Kayama et al. 2009), and in all compositions between oligoclase and andesine in the shocked samples from the Ries impact structure (Germany) investigated by Stöffler (1967). These planar microstructures formed at shock pressures of 18–30 GPa, as confirmed by shock experiments (Ostertag 1983). Stöffler (1967) observed that isotropization of plagioclase seems to begin along thin deformation lamellae (1–8 μm), especially in twinned crystals, suggesting that shock transformation into diaplectic glass is also, at least to some extent, crystallographically controlled.

Advances in conventional methods and the development of new analytical techniques have allowed further detailed characterization of shocked feldspar, even in the case of complex crystal structures in low shock regimes (e.g., Jaret et al. 2014, 2018; Pickersgill et al. 2015). These studies demonstrate that feldspar is an important mineral group to be considered in the study of shock metamorphism and hypervelocity impacts in general.

Here, we present a study of shocked plagioclase feldspar, focusing on information provided by electron backscatter diffraction (EBSD), on a sample of shocked metagranite in a shatter cone from the Manicouagan impact structure, Canada (e.g., White 1993). We have identified and characterized the discrete features, namely amorphous lamellae that result from shock metamorphism of plagioclase, and we discuss their formation mechanism during the impact cratering process. Finally, we suggest that, due to the limitations of analytical instrumentation applied in some previous studies, microstructures reported as PDFs or impact‐induced microtwins in plagioclase should be reconsidered, as they are difficult to be correctly identified with the methods used at that time, and the interpretation on their formation process should be reevaluated.

## Methods

**Fig. 1:**
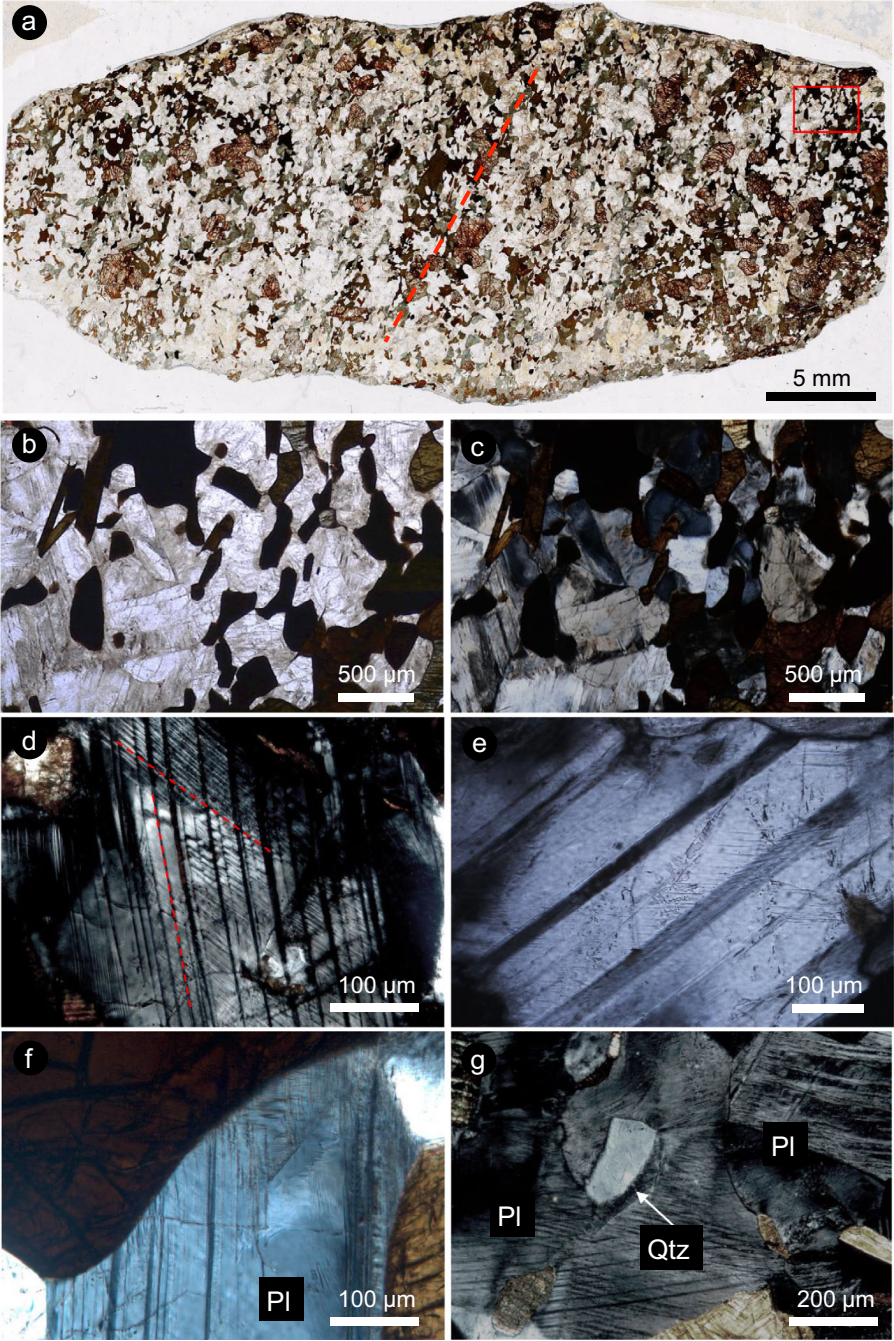
Shatter cone sample WMM‐102A‐64C1 from the Manicouagan impact structure. a) Plane polarized light mosaic of the investigated thin section. The striated surface of the shatter cone corresponds to the upper edge of the section. Note the weak foliation delineated by the mafic and opaque minerals (mostly amphibole and garnet). The foliation direction is marked with a red dashed line). b) Detail of the sample as seen in plane polarized light. Note the dense arrangement of planar microstructures in plagioclase grains. Optical microscope, plane polarized light. c) Same area of the thin section between crossed polarizers, to highlight twins, isotropic domains, and domains with reduced birefringence in plagioclase. Optical microscope, crossed polarizers. d) Two sets of crosscutting twin lamellae in plagioclase, highlighted by red dashed lines. Optical microscope, crossed polarizers. e) Plagioclase grain showing strongly reduced birefringence of one twin set (interference color dark gray) and the presence of thin lamellae in the other twin set. Optical microscope, crossed polarizers. f) Planar microstructures in plagioclase (Pl) that seem to originate from the grain boundary between plagioclase and a mafic mineral. Optical microscope, crossed polarizers. g) Planar microstructures, likely twins, in plagioclase (Pl) crystals with reduced birefringence and isotropic domains in contact with birefringent quartz (Qtz) crystal. Optical microscope, crossed polarizers. (Color figure can be viewed at wileyonlinelibrary.com.)

A 30 µm thick, feldspar‐rich, polished petrographic thin section (Fig. 1a) from sample WMM‐102A‐64C1, a shatter cone from the central uplift of the Manicouagan impact structure, was selected for this study. The thin section was cut normal to the shatter cone surface and to the striation. The section was initially surveyed by optical microscopy and scanning electron microscopy (SEM) to identify regions of the sample where shock microstructures in feldspar are better developed. SEM was performed at the Department of Lithospheric Research at the University of Vienna (Austria) with an FEI Inspect S50 SEM, operated at 10–15 kV, variable spot size depending on the magnification and the required analysis, and at 10 mm working distance.

The thin section was later prepared for EBSD by mechanical polishing using 1 and 0.3 µm alumina suspended in distilled water for 10 min each, followed by up to 4 h on colloidal silica in an NaOH suspension. The sample was then coated with a 5–7 nm conductive layer of carbon. EBSD data were collected using a Zeiss Sigma variable pressure field emission gun (VP‐FEG)‐SEM equipped with an Oxford Instruments NordlysMax2 EBSD detector at the ISSAC microscopy facility at the University of Glasgow (UK). Operating conditions during EBSD data acquisition were 20 kV accelerating voltage, a 4 nA beam current, and a tilt angle of 70°. EBSD maps were collected with a step size of 0.2–0.4 µm and a working distance of 10–15 mm on selected plagioclase crystals containing shock microstructures, as seen under the optical microscope. Static and dynamic background corrected Kikuchi patterns were collected from each pixel in the map with a 4 × 4 binning, an exposure time between 35 and 80 ms, and a frame average between 1 and 3, depending on the area. The bytownite match unit was used to index plagioclase grains and the mean angular deviation (MAD; a measure of the quality of the pattern indexing where <1 represents a good match) was between 0.58 and 0.87 for the Kikuchi patterns in all the acquired EBSD maps. Crystallographic information was extracted from the EBSD data using the Channel 5 software package. The EBSD data were noise reduced using a single wildspike correction followed by an iterative six point nearest neighbor correction. This procedure helps define grains without generating significant artifacts (e.g., Bestmann and Prior 2003). Various types of maps were generated from the EBSD data including Band Contrast, Euler, inverse pole figure (IPF; *x*,*y*,*z*), and grain reference orientation distribution (GROD) angle. Band Contrast maps visualize the electron backscatter pattern quality across a map, where darker grayscale indicates reduced pattern quality. IPF and Euler maps are two approaches to visualize the orientation of a crystal relative to the plane of the sample section through color. GROD angle maps indicate the variation in internal misorientation relative to the average orientation of the grain (where a grain is defined as a <10° internal misorientation) with increasing degrees of internal misorientation relative to the average indicated by progressively warmer colors (yellow to ‐red). Euler, IPF, and GROD angle maps are overlain onto the band contrast maps with semitransparency (i.e., in all maps, lower band contrast can be seen as a darkening of the Euler/IPF/GROD angle colors).

The orientation of the amorphous lamellae was graphically related to rational crystallographic orientations, by comparing the possible orientation of the poles of the amorphous lamellae (normal to the trace of the lamellae in the section) with the orientation of the more common twin laws and to the occurrence of “PDFs” in plagioclase as reported in the literature (e.g., Stöffler 1967). Additional measurements with the Universal‐stage (U‐stage) were performed on selected domains in both quartz, to determine the orientation of PDF sets according to the traditional method (e.g., Langenhorst 2002), and plagioclase, to determine the dip angle between the investigated lamellae and the surface of the thin section, reducing the number of possible orientations that are consistent with the trace of the lamellae in EBSD maps. Crystallographic data are presented in Miller indices (Miller‐Bravais indices in the case of quartz), with the following convention: (h,k,l) for poles and {h,k,l} for families of planes. In addition, major and minor element composition of the investigated plagioclase grains was analyzed with a CAMECA SXFive field‐emission electron microprobe (EPMA), equipped with four WDS and one EDS detectors at the Department of Lithospheric Research, University of Vienna, Austria. Operating conditions were 15 kV accelerating voltage, 20 nA beam current, defocused beam diameter of 5 μm size, and PAP correction procedure for data reduction (Pouchou and Pichoir 1991). Natural and synthetic standards were used for calibration. Detection limits for all the considered elements are <400 µg g^−1^.

## Results

### Petrographic Features in Plagioclase

The sample used in this study is a garnet‐bearing metagranite, which exhibits a weak foliation marked by alignment of mafic minerals (Fig. 1a). The major minerals, in order of decreasing abundance, are plagioclase (Or_<5_An_23_, oligoclase), garnet, hornblende, biotite, quartz, and accessory titanite and ilmenite. Optical microscope observations reveal that overall, the sample appears moderately shocked, displaying intense fracturing of garnet, kinking of biotite, and the widespread presence of planar microstructures in plagioclase and quartz. These shock features are homogeneously distributed across the thin section, and there is no gradient in the abundance of shock features from the surface of the shatter cone inward.

Most quartz grains display a single set of decorated PDFs, oriented parallel to the $\left\{10\bar{1}3\right\}$ orientation, as determined with the U‐stage. The mineral containing the most abundant shock features as seen with the optical microscope is plagioclase, which shows a range of lamellae, including straight penetrating planar microstructures, and locally bent lamellae (Fig. 1b,c). Such lamellae are widespread and present in several orientations, and they appear dark when observed between crossed polarizers, suggesting isotropization, and show a wide range of thicknesses (Fig. 1d,e). Locally, two crosscutting twin sets occur. It seems that in some plagioclase crystals, the density of such lamellae is increased in areas where the plagioclase is in contact with other mineral grains, such as quartz (Fig. 1f–g).

**Fig. 2:**
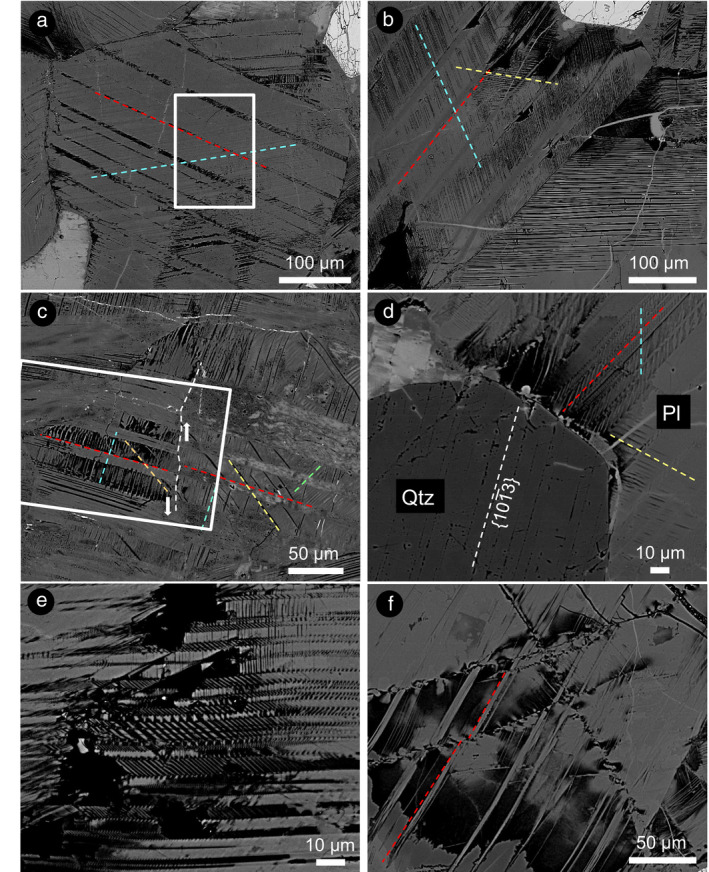
BSE‐SEM images of planar microstructures in plagioclase. a) Alternating thick (approximately 30 µm; dashed red line) and thin (10 µm) twins. The ~10 µm set is characterized by a set of thin lamellae (dashed light blue line), which are oriented at a high angle to the main twin set. The area investigated with EBSD is marked with a solid white box. b) Twin system with the thick twins (dashed red line) characterized by sets of thin lamellae (light blue line) normal to the thick lamellae in a plagioclase crystal. In the lower right of the image, another plagioclase crystal shows only one set of thin lamellae extending from the grain margin. c) Crystal with alternate twins containing secondary lamellae. Twins are displaced along a crack (marked with dashed white line, whose shear sense is indicated with arrows). Note that the twins with no lamellae on the right hand side of the crack display evidence for having undergone incipient melting and devitrification, with flow fabric and crystallization of fine‐grained, K‐rich phases. The area investigated with EBSD is marked with a solid white box. d) Plagioclase (Pl) adjacent to quartz (Qtz), showing higher density of lamellae next to the upper margin of the quartz grain. Note that the quartz grain presents one obvious set of decorated PDFs. e) Complex arrangement of mutually crosscutting sets of lamellae within a plagioclase crystal. f) Crystal distortion along a parallel fracture set (likely planar fractures), apparently accommodating the formation of lamellae in specific areas of the grain. These fractures displace thin lamellae in plagioclase. (Color figure can be viewed at wileyonlinelibrary.com.)

Due to differential polishing, these lamellae are visible in some cases also in secondary electron (SE) and backscattered electron (BSE)‐SEM images (Fig. 2). Some sets of lamellae can only be identified due to the presence of a second set, crosscutting, or included within the major one (Fig. 2a). These additional sets of lamellae are thin (1–2 µm), generally straight over the crystal length, and locally present in multiple sets (Fig. 2b). In some crystals, such secondary sets are present within one set of thick lamellae (10–20 µm), alternating with another set of comparable thickness, but free from secondary lamellae (Fig. 2c). Some of these thick lamellae appear to have experienced impact melting (as indicated by the presence of a flow fabric) and subsequently recrystallized to a fine‐grained mosaic (Fig. 2c). The crystals are locally crosscut by fractures that generate small offsets, identifiable by the displacement of the lamellae (Fig. 2c). The density of such lamellae is locally varied; they are particularly concentrated close to the boundary between plagioclase and other mineral phases with a different shock impedance (i.e., quartz; Fig. 2d). An example of a high density of lamellae is represented by the presence of crosscutting sets of lamellae (Fig. 2e). These lamellae are locally crosscut by sets of subparallel fractures that dislocate the lamellae, causing an apparent lattice distortion (Fig. 2f). However, SE and BSE images do not provide information on the crystallinity of the investigated phases, but EBSD does (see below).

### EBSD Analysis of Plagioclase

**Fig. 3:**
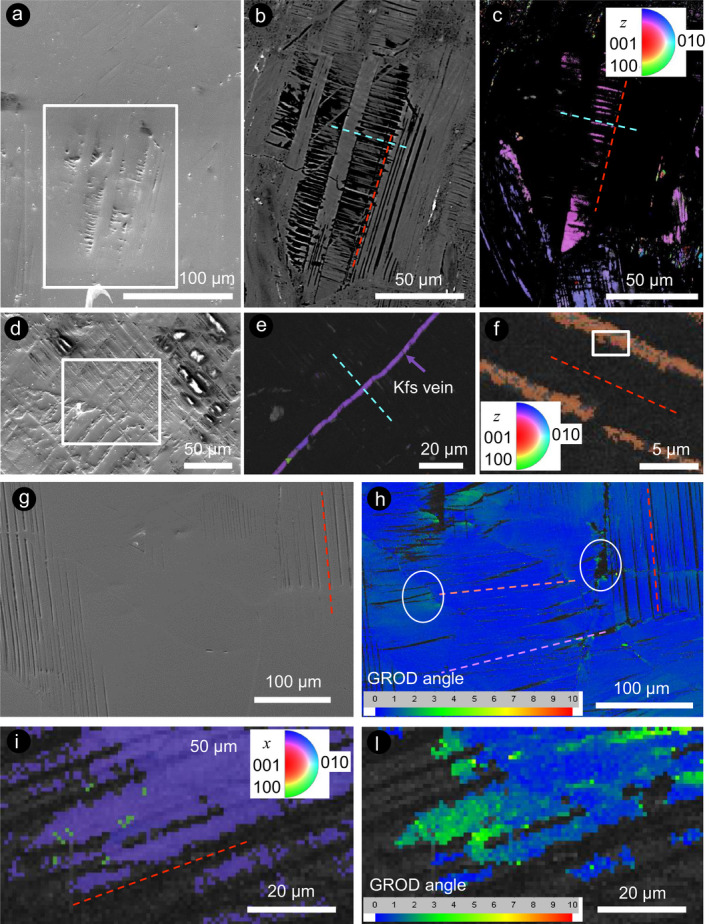
EBSD results from selected areas of plagioclase crystals containing planar microstructures. a) SE‐SEM image, (b) BSE‐SEM image, and (c) inverse pole figure (IPF) map of the area in Fig. 2c, rotated 90° from original data collection orientation. The twin characterized by the presence of secondary lamellae shows residual crystallinity, whereas the other twins are completely amorphous. The amorphous twins appear black in the IPF map, because they produced no Kikuchi patterns, and this effect is not due to differential polishing (a). d) SE‐SEM image and (e) IPF map of a plagioclase crystal, where the differential polishing reveals two sets of crosscutting microstructures, both largely amorphous in the EBSD map, and crosscut by a perfectly crystalline K‐feldspar vein (e). f) IPF map of a plagioclase crystal that displays a set of parallel alternating crystalline and amorphous lamellae. g) Secondary electron (SE)‐SEM image and (h) GROD angle map of a crystal with lamellae that are amorphous (black in the image) or have reduced band contrast (darker than the main crystal). Colors denote the degree of internal misorientation angle, ranging from 0° (blue), corresponding to the orientation of the host crystal, to 2–6° misorientation (green‐yellow), with respect to the average crystallographic orientation of the host crystal. Note the lattice deformation next to the cracks (white circles). i) IPF map, and (l) GROD angle map of another plagioclase crystal with a set of lamellae, which are characterized by progressively lower band contrast than that of the host crystal up to complete amorphization (left part of each image). Note the lattice distortion in correspondence of the amorphous lamellae (l). (Color figure can be viewed at wileyonlinelibrary.com.)

**Fig. 4:**
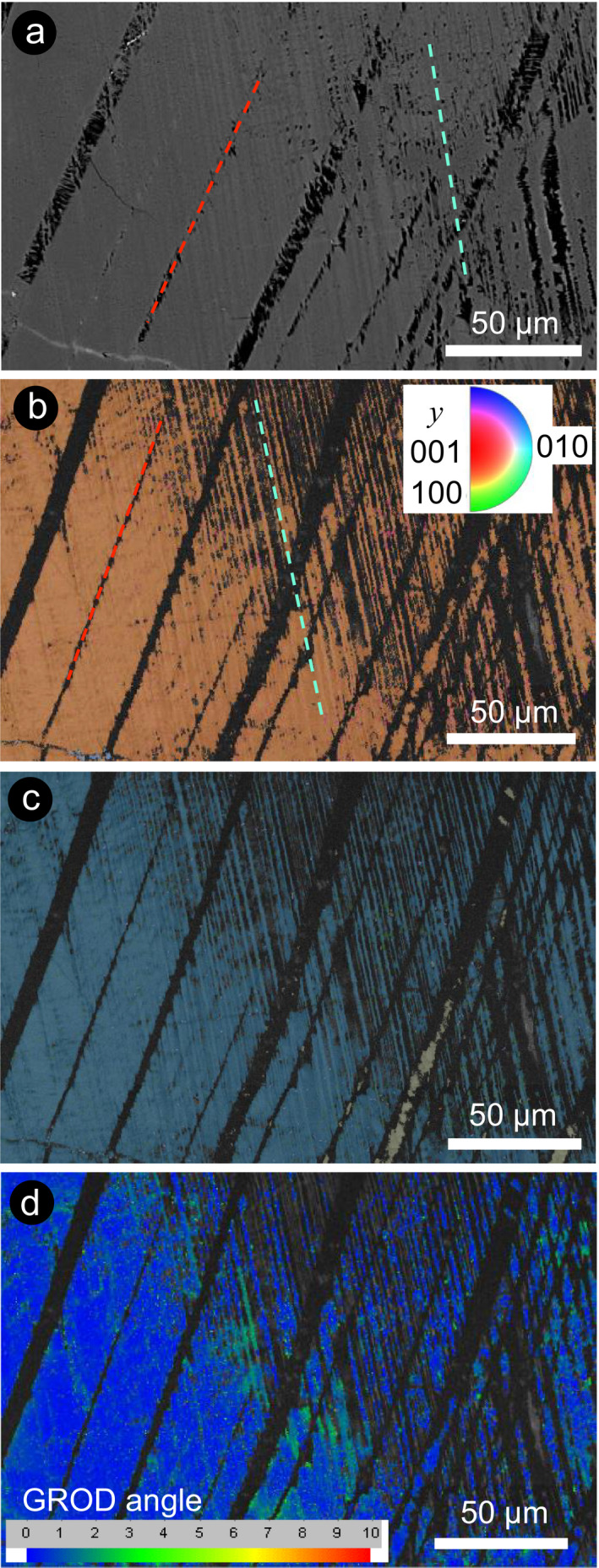
EBSD results from a plagioclase crystal containing two sets of lamellae. a) BSE‐SEM image, (b) IPF map, (c) Euler map, and (d) grain reference orientation difference (GROD) angle map of the area in Fig. 2a. Note that the image has been rotated 90° clockwise with respect to Fig. 2a. The two sets of amorphous lamellae (black in the IPF map and delineated by dashed lines) are characterized by different thicknesses (approximately 8 µm and <2 µm, respectively), spacing, and orientations. Thick (parallel to red line) and thin (parallel to light blue line) lamellae are apparent. Alternate thick lamellae have regions that are amorphous (black) and regions that are crystalline (khaki green in the Euler map). The misorientation profiles across the main crystal (blue) into the thick lamellae (khaki green) back into the main crystal (blue) reveal a 180° rotation around the {010} planes, suggesting that these thick lamellae were once igneous twins. Alternate thin lamellae exhibit a progression from a reduced band contrast in the bottom left part of the image to complete amorphization (black subvertical lamellae) in the top right part of the image. (Color figure can be viewed at wileyonlinelibrary.com.)

To complement the optical microscopy and SEM work, EBSD was used to investigate the presence of amorphous lamellae in plagioclase at a higher spatial resolution than that achieved by optical microscopy. EBSD maps of plagioclase containing shock‐induced planar microstructures show that many of the alternating lamellae are likely amorphous (i.e., they do not produce Kikuchi patterns) or have reduced crystallinity (i.e., they are characterized by low band contrast), despite being well polished (Figs. 3 and 4). Thick lamellae (~30 µm), likely magmatic twins, which are common in this lithology, are either completely amorphous or contain an internal set of thin lamellae (<5 µm), which are amorphous and oriented at high angle to the main set of lamellae (Fig. 3a–f). Locally, shock features are separated by cracks (Figs. 3a–c and 3e). In the case of Figs. 2c and 3a–c, the crack separates domains that exhibit distinct shock effects on either side of the fracture. This suggests that the crack predated the impact event, generating an anisotropy within the grain that resulted in the minerals on each side of the crack experiencing different peak shock pressures. The lamellae are slightly offset on either side of the crack (~10 µm), but on the one side, the lamellae are crystalline, and on the other side, they are either completely amorphous or display flow fabric (Figs. 2c and 3b,c).

Locally, it is still possible to identify the crystallographic orientation of some domains with low crystallinity (Figs. 3e–f). Thin amorphous lamellae locally occur only in a portion of a crystal, apparently determining local lattice distortion, as shown in the GROD maps (Figs. 3g–l). In some cases, the amorphization of pre‐existing twins (i.e., thick sets of lamellae) is not complete and the twin law can be constrained, as in the crystal shown in Fig. 4, where the major (thick) set is consistent with twins generated by rotation with respect to the (010) orientation (albite‐twinning). The amorphization of the thin lamellae is not consistent across the grain (i.e., the top right part of the image shows amorphous lamellae, the bottom left part shows crystalline lamellae of reduced band contrast). Notably, in the area where the thin set of lamellae are crystalline, they do not demonstrate a twin relationship with the host crystal, as demonstrated by the fact that both lamellae and host have the same color in the Euler map (i.e., same crystallographic orientation).

## Discussion

### Localization of Shock‐Amorphization

As mentioned earlier, the common definition of PDFs in quartz is thin (<200–300 nm), closely spaced (<1 μm), amorphous lamellae, which develop along rational crystallographic planes (e.g., von Engelhardt and Bertsch 1969; Stöffler and Langenhorst 1994). Although PDFs in quartz occur frequently in impactites, their formation mechanism is still debated (e.g., Goltrant et al. 1992; Trepmann 2008). Optically isotropic sets of lamellae in shocked plagioclase with similar characteristics to PDFs in quartz have also been described as PDFs. In the investigated sample, other than the thick isotropic lamellae, which represent amorphized twins, we also observe thin lamellae that locally resemble PDFs, as already proposed by Ferrière and Osinski (2013).

**Fig. 5:**
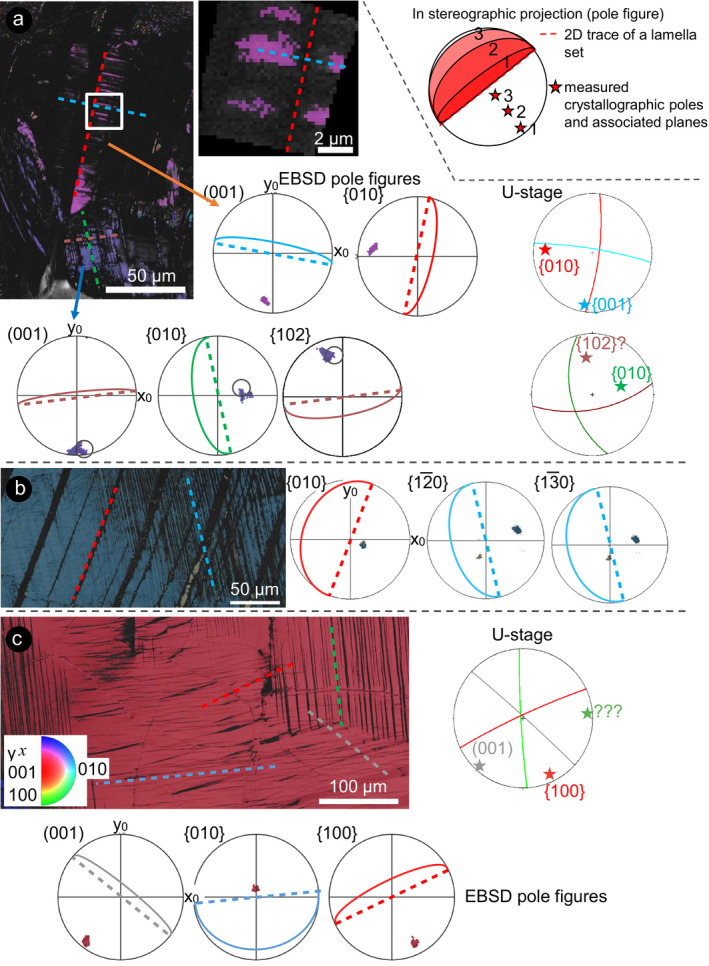
Attempt to empirically index the crystallographic orientation of the amorphous lamellae in plagioclase feldspar. Note that all the plots are color coordinated. In the inset, a schematic representation of the theory behind the transition from an IPF map to pole figures is provided. a) IPF map and pole figures corresponding to key crystallographic planes in the area of Fig. 3a. A comparison with the stereographic projection of selected sets as determined with the U‐stage is provided. For the crystal with orientation colored pink in the map, the orientation of the major twin system (red dashed line) is consistent with {010} (red dotted line) as symmetry plane, and that of the lamellae present only in one twin (blue dashed line) with symmetry plane {001} (blue dotted line). A similar relationship between twins seems to be displayed by a neighboring crystal (violet, only part of the data cluster marked with a circle; with twins marked by orange and green dashed lines), compared with the U‐stage data. b) Euler map and pole figures of the area in Fig. 4. The thick set of lamellae corresponds to {010}, whereas the thin set is likely consistent with $\left\{1\bar{3}0\right\}$. c) IPF map and pole figures corresponding to the area in Fig. 3g. The set of lamellae with reduced band contrast (blue dashed line) is consistent with {010} (blue dotted line), but was not visible using the U‐stage. The secondary set marked with red dashed line corresponds to {100}. The set of cracks (gray dashed line) is oriented parallel to (001). The major set of lamellae (green dashed line), visible also with the U‐stage, does not match any of the notable crystallographic orientations expected in plagioclase. (Color figure can be viewed at wileyonlinelibrary.com.)

These thin lamellae have been interpreted as microtwins, possibly induced or amorphized by shock, in earlier literature (e.g., White 1993; Jaret et al. 2014). However, in some regions within the crystals, these lamellae are not completely amorphous and show only reduced EBSD band contrast. These lamellae propagate into totally amorphous regions (i.e., with no detectable band contrast). The fact that some regions of the lamellae are crystalline allows us to determine their crystallographic orientation. EBSD characterization of these partially to fully crystalline regions of lamellae reveals that they do not show a twin relationship with the host crystal and instead share the same crystallographic orientation as the host crystal (Figs. 4 and 5). The identification of twins in plagioclase can be successfully performed with EBSD, but both individuals of a set must be crystalline to be structurally characterized (e.g., Xu et al. 2016). The orientation of shock‐induced, planar amorphous lamellae is commonly determined using the U‐stage, because it allows the evaluation of the 3D orientation of any planar microstructure within a given crystal (e.g., Langenhorst 2002). However, this technique is particularly challenging in plagioclase owing to its complex optical properties deriving from the solid solution of the feldspar group (e.g., Stöffler 1967). In our case, the presence of amorphous twin sets hampers the use of both the U‐stage (i.e., it is impossible to determine the optical axes) and EBSD (i.e., amorphous individuals in twin sets cannot be indexed) to determine the orientation of the lamellae. However, a combination of these two techniques, as described in the Methods section, allows us to determine the most probable crystallographic orientation(s) of the investigated lamellae (Fig. 5). We compared the possible orientation of the poles of the amorphous lamellae, determined as great circles normal to the trace of the lamellae in SEM images, with the most common orientations of twins and PDFs in plagioclase, supporting the interpretation based on the U‐stage orientation data.

Considering the similarity in the observations of PDFs in quartz and amorphous lamellae in plagioclase, which resemble microtwins, it could be inferred that the formation mechanism of PDFs is similar for quartz and members of the feldspar family. The major difference in quartz, with respect to plagioclase, would be that PDFs in quartz form along orientations that are not known for twin development (e.g., Gault 1949), whereas the most common PDF orientations in plagioclase (e.g., Stöffler 1967) are those that are also exploited by twins. Even though there is no evidence of shock‐induced twinning so far, twinning might still be an important additional effect of shock. The distribution of symmetrically equivalent PDF sets in quartz seems to reflect hexagonal rather than trigonal symmetry (e.g., Stöffler and Langenhorst 1994), suggesting quartz twinning according to the Dauphiné twin law. Dauphiné twins have been demonstrated to form mechanically at low pressure and by retrogression from β‐ to α‐quartz (e.g., Wenk et al. 2005; Trepmann and Spray 2005; Menegon et al. 2011). This makes their presence compatible with the shock conditions encountered by a quartz crystal, which may contain multiple sets of PDFs. In addition, the presence of Brazil twins induced by low shock and in relation with the amorphization of one twin in quartz was also suggested by Goltrant et al. (1992) and Fazio et al. (2018) as a precursor for amorphization. Therefore, we cannot exclude that in plagioclase also a twin mechanism precedes and favors the localization of shock amorphization.

### Shock Evolution of Plagioclase

For the investigated sample, our suggested sequence of deformation steps in plagioclase is:

1. Pre‐impact processes:
  - Plagioclase crystallization and primary/magmatic twinning.
  - Secondary (i.e., glide) twinning due to tectonic deformation of the host rock.
2. Shock deformation:
  - Likely collapse/failure of specific crystallographic planes due to compression at the front of the shock wave, and preferentially at the crystal margin in contact with minerals of other shock impedance or due to reflection of the shock wave at the grain boundary, forming lamellae consisting of diaplectic glass.
  - Possibly subsequent amorphization of selected pre‐impact twins, due to adiabatic decompression at the tail of the shock wave (e.g., Langenhorst 2000).
3. Post–impact deformation:
  - Formation of additional fractures, circulation of fluids, crystallization of K‐feldspar along cracks, and local devitrification and recrystallization of impact glass with feldspathic composition.

Whether or not individual crystals have been shock amorphized is likely controlled by their crystallographic orientation with respect to the local shock wave, as already suggested by Stöffler (1967) and references therein. The local orientation of the shock wave is variable because it is scattered as a result of interaction with minerals of different shock impedances and microstructural characteristics than that of plagioclase. Also the possibly different orientation of neighboring plagioclase crystals may cause scattering/refraction/reflection of the shock wave. The crystallographic orientation of the closely spaced, twin‐like amorphous lamellae is, on the other hand, determined by the shock intensity and the characteristics of the plagioclase lattice. Viscous–plastic deformation of plagioclase is known to activate specific slip systems, with (010) as a common slip plane (e.g., Stünitz et al. [2003] and references therein). Additionally, brittle deformation tends to be localized along orientations that are known as cleavage planes (e.g., Brown and Macaudière 1984). We can assume that for high strain rates, as is the case for shock metamorphism, a similar localization mechanism is activated and specific crystallographic orientations are more prone than others to undergo failure. This is the formation mechanism proposed by Goltrant et al. (1992) for PDFs in quartz. The characteristics of the plagioclase lattice, in contrast with that of quartz, determine not only the periodicity of strain microstructures but also the common directions of structural weakness, along which deformation tends to be localized (Brown and Macaudière 1984). This would be consistent with the pattern of alternating amorphous lamellae being similar to the one produced by twinning, without requiring the formation of twins as precursor for amorphization. In quartz, the different crystallographic orientations of PDFs (i.e., planes that underwent failure) are correlated with the intensity of the shock pressure (e.g., Stöffler and Langenhorst 1994). The same should be applied for plagioclase and, thus, we can imagine in the future to use planar shock features in plagioclase as a shock barometer.

In the case of the investigated sample, we do not have sufficient constraints (e.g., an EBSD map of the complete thin section, which was hampered by the difficulty to obtain homogenous polishing and the time required to reach the desired resolution) to establish a clear relation between individual crystallographic orientations of plagioclase or lamellae within plagioclase and probable shock wave propagation direction or intensity. Dedicated experiments should be able to establish such a correlation, using the amorphization of twin‐like lamellae as shock barometers. The results are presumably in the relatively low shock regime, as suggested by previous works (e.g., Jaret et al. 2014) and by the coexistence of shocked quartz with mostly just one PDF set along $\left\{10\bar{1}3\right\}$ in the investigated sample.

## Conclusions

The investigated sample, collected in the central uplift of the Manicouagan impact structure, Canada, contains abundant shocked plagioclase. The most common shock feature is the presence of several parallel sets of lamellae of variable thicknesses in plagioclase that are either amorphous or exhibit reduced birefringence and crystallinity. The sets of thicker (>20 µm) lamellae likely originated as magmatic twins, whereas the thinner sets (<5 µm) consist of closely spaced amorphous lamellae, similar to those that have been interpreted in the literature as both shock‐induced microtwins (e.g., Dworak 1969) and PDFs (Dressler 1990). Our microstructural and EBSD observations suggest a two‐stage shock amorphization process: (i) formation of closely spaced amorphous lamellae due to shock compression and (ii) amorphization of selected pre‐existing coarse‐grained twins, presumably due to shock relaxation. This second stage is likely controlled by the crystallographic orientation of the twin set, as suggested by Stöffler (1967) and Dworak (1969). The use of EBSD allows for complete characterization of such lamellae. Our observations on the thin amorphous lamellae suggest that they did not form as twins, but rather along specific crystallographic directions that collapse in the high strain rate regime, similar to the formation mechanism proposed for PDFs in quartz (e.g., Goltrant et al. 1992). Therefore, the formation of such amorphous lamellae in plagioclase lends further support to the suggested mechanism for PDFs in quartz, perhaps providing a formation mechanism that could also be applicable to other silicate minerals.

## Editorial Handling

Dr. W. Uwe Reimold

## References

[maps13494-bib-0001] Bestmann M. and Prior D. J. 2003 Intragranular dynamic recrystallization in naturally deformed calcite marble: Diffusion accommodated grain boundary sliding as a result of subgrain rotation recrystallization. Journal of Structural Geology 25:1597–1613.

[maps13494-bib-0002] Binns R. A. 1967 Stony meteorites bearing maskelynite. Nature 213:1111–1112.

[maps13494-bib-0003] Brown W. L. and Macaudière J. 1984 Microfracturing in relation to atomic structure of plagioclase from a deformed meta‐anorthosite. Journal of Structural Geology 6:579–586.

[maps13494-bib-0004] Chen M. and El Goresy A. 2000 The nature of maskelynite in shocked meteorites: Not diaplectic glass but a glass quenched from shock‐induced dense melt at higher pressures. Earth and Planetary Science Letters 179:489–502.

[maps13494-bib-0005] Dressler B. 1990 Shock metamorphic features and their zoning and orientation in the Precambrian rocks of the Manicouagan Structure, Quebec, Canada. Tectonophysics 171:229–245.

[maps13494-bib-0006] Dworak U. 1969 Stoßwellenmetamorphose des Anorthosits vom Manicouagan Krater, Québec, Canada. Contributions to Mineralogy and Petrology 24:306–347. In German.

[maps13494-bib-0007] Engelhardt W. von , and Bertsch W. 1969 Shock induced planar deformation structures in quartz from the Ries Crater, Germany. Contributions to Mineralogy and Petrology 20:203–234.

[maps13494-bib-0008] Fazio A. , Pollok K. , and Langenhorst F. 2018 Experimental evidence for mechanical Brazil twins as an indicator of low‐pressure shock metamorphism (<17.5 GPa). Geology 46:787–790.

[maps13494-bib-0009] Ferrière L. and Brandstätter F. 2015 What is maskelynite? Back to the original description and thin sections in which it was first described. 78th Annual Meeting of the Meteoritical Society (abstract #. 5184).

[maps13494-bib-0010] Ferrière L. and Osinski G. R. 2013 Shock metamorphism In Impact cratering: Processes and products, edited by Osinski G. R. and Pierazzo E. Chichester, UK: Wiley‐Blackwell pp. 106–124.

[maps13494-bib-0011] Fritz J. , Greshake A. , and Stöffler D. 2005 Micro‐Raman spectroscopy of plagioclase and maskelynite in Martian meteorites: Evidence of progressive shock metamorphism. Antarctic Meteorite Research 18:96–116.

[maps13494-bib-0012] Fritz J. , Wünnemann K. , Greshake A. , Fernandes V. A. S. M. , Boettger U. , and Hornemann U. 2011 Shock pressure calibration for lunar plagioclase (abstract #1169). 42nd Lunar and Planetary Science Conference. CD‐ROM.

[maps13494-bib-0013] Fritz J. , Assis Fernandes V. , Greshake A. , Holzwarth A. , and Böttger U. 2019 On the formation of diaplectic glass: Shock and thermal experiments with plagioclase of different chemical compositions. Meteoritics & Planetary Science 54:1533–1547.

[maps13494-bib-0014] Gault H. R. 1949 The frequency of twin types in quartz crystals. American Mineralogist 34:142–162.

[maps13494-bib-0015] Gillet P , Chen M. , Dubrovinsky L. , and El Goresy A. 2000 Natural NaAlSi_3_O_8_‐Hollandite in the shocked Sixiangkou meteorite. Science 287:1633–1636. 10698734 10.1126/science.287.5458.1633

[maps13494-bib-0016] Goltrant O. , Cordier P. , and Doukhan J.‐C. 1991 Planar deformation features in shocked quartz; a transmission electron microscopy investigation. Earth and Planetary Science Letters 106:103–115.

[maps13494-bib-0017] Goltrant O. , Leroux H. , Doukhan J.‐C. , and Cordier P. 1992 Formation mechanisms of planar deformation features in naturally shocked quartz. Physics of the Earth and Planetary Interiors 74:219–240.

[maps13494-bib-0018] Grieve R. A. F. , Langenhorst F. , and Stöffler D. 1996 Shock metamorphism of quartz in nature and experiment: II. Significance in geoscience. Meteoritics & Planetary Science 31:6–35.

[maps13494-bib-0019] Huffman A. R. and Reimold W. U. 1996 Experimental constraints on shock‐induced microstructures in naturally deformed silicates. Tectonophysics 256:165–217.

[maps13494-bib-0020] Jaret S. J. , Kah L. C. , and Harris R. S. 2014 Progressive deformation of feldspar recording low‐barometry impact processes, Tenoumer impact structure, Mauritania. Meteoritics & Planetary Science 49:1007–1022.

[maps13494-bib-0021] Jaret S. J. , Woerner W. R. , Phillips B. L. , Ehm L. , Nekvasil H. , Wright S. P. , and Glotch T. D. 2015 Maskelynite formation via solid‐state transformation: Evidence of infrared and X‐ray anisotropy. Journal of Geophysical Research 120:570–587.

[maps13494-bib-0022] Jaret S. J. , Johnson J. R. , Sims M. , DiFrancesco N. , and Glotch T. D. 2018 Microspectroscopic and petrographic comparison of experimentally shocked albite, andesine, and bytownite. Journal of Geophysical Research 123:1701–1722.

[maps13494-bib-0023] Kayama M. , Gucsik A. , Nishido H. , Ninagawa K. , and Tsuchiyama A. 2009 Cathodoluminescence and Raman spectroscopic characterization of experimentally shocked plagioclase In Micro‐Raman spectroscopy and luminescence studies in the Earth and planetary sciences. Proceedings of the International Conference Spectroscopy 2009. AIP Conference Proceedings 1163: 86–95.

[maps13494-bib-0024] Langenhorst F. 2002 Shock metamorphism of some minerals: Basic introduction and microstructural observations. Bulletin of Czech Geological Survey 77:265–282.

[maps13494-bib-0025] Langenhorst F. , Joreau P. , and Doukhan J. C. 1995 Thermal and shock metamorphism of the Tenham chondrite: A TEM examination. Geochimica et Cosmochimica Acta 59:1835–1845.

[maps13494-bib-0026] Menegon L. , Piazolo S. , and Pennacchioni G. 2011 The effect of Dauphiné twinning on plastic strain in quartz. Contributions to Mineralogy and Petrology 161:635–652.

[maps13494-bib-0027] Ostertag R. 1983 Shock experiments on feldspar crystals. Proceedings of the 14th LPSC. Journal of Geophysical Research 88:B364–B376.

[maps13494-bib-0028] Papike J. , Taylor L. , and Simon S. 1991 Lunar minerals In Lunar source‐book, edited by Heiken G. H., Vaniman D. T., and French B. M. New York: Cambridge University Press pp. 121–181.

[maps13494-bib-0030] Pittarello L. , Schulz T. , Koeberl C. , Hoffmann J. E. , and Münker C. 2013 Petrography, geochemistry, and Hf‐Nd isotope evolution of drill core samples and target rocks from the El'gygytgyn impact crater, NE Chukotka, Arctic Russia. Meteoritics & Planetary Science 48:1160–1198. 10.1111/maps.12146 PMC4461123 26074719

[maps13494-bib-0031] Pouchou J.‐L. and Pichoir F. 1991 Quantitative analysis of homogeneous or stratified microvolumes applying the model “PAP.” In Electron probe quantitation, edited by Heinrich K. F. J. and Newbury D. E. Boston, MA: Springer pp. 31–75.

[maps13494-bib-0032] Sims M. , Jaret S. J. , Carl E.‐R. , Rhymer B. , Schrodt N. , Mohrholz V. , Smith J. , Konopkova Z. , Liermann H.‐P. , Glotch T. D. , and Ehm L. 2019 Pressure‐induced amorphization in plagioclase feldspars: A time‐resolved powder diffraction study during rapid compression. Earth and Planetary Science Letters 507:166–174.

[maps13494-bib-0033] Sims M. , Jaret S. J. , Johnson J. R. , Whitaker M. L. , and Glotch T. D. 2020 Unconventional high‐pressure Raman spectroscopy study of kinetic and peak pressure effects in plagioclase feldspars. Physics and Chemistry of Minerals 47:1–10.

[maps13494-bib-0034] Stöffler D. 1967 Deformation und Umwandlung von Plagioklas durch Stoßwellen in den Gesteinen des Nördlinger Ries. Contributions to Mineralogy and Petrology 16:51–83. In German.

[maps13494-bib-0035] Stöffler D. and Langenhorst F. 1994 Shock metamorphism of quartz in nature and experiment: I. Basic observation and theory. Meteoritics & Planetary Science 29:155–181.

[maps13494-bib-0036] Stöffler D. , Hamann C. , and Metzler K. 2018 Shock metamorphism of planetary silicate rocks and sediments: proposal for an updated classification system. Meteoritics & Planetary Science 53:5–49.

[maps13494-bib-0037] Stünitz H. , Fitz Gerald J. D. , and Tullis J. 2003 Dislocation generation, slip systems, and dynamic recrystallization in experimentally deformed plagioclase single crystals. Tectonophysics 372:215–233.

[maps13494-bib-0038] Trepmann C. A. 2008 Shock effects in quartz: compression versus shear deformation—an example from the Rochechouart impact structure, France. Earth and Planetary Science Letters 267:322–332.

[maps13494-bib-0039] Trepmann C. A. and Spray J. G. 2005 Planar microstructures and Dauphiné twins in shocked quartz from the Charlevoix impact structure, Canada. In Large meteorite impacts III, edited by Kenkmann T., Hörz F., and Deutsch A. *Geological Society of America Special Paper* 384:315–328.

[maps13494-bib-0040] Tschermak G. 1872 Die Meteoriten von Shergotty und Gopalpur. Sitzungsberichte der Kaiserlichen Akademie der Wissenschaften zu Wien LXV 1:122–146. In German.

[maps13494-bib-0041] Wenk H. R. , Lonardelli I. , Vogel S. C. , and Tullis J. 2005 Dauphiné twinning as evidence for an impact origin of preferred orientation in quartzite: An example from Vredefort, South Africa. Geology 33:273–276.

[maps13494-bib-0042] White J. C. 1993 Shock‐induced amorphous textures in plagioclase, Manicouagan, Quebec, Canada. Contributions to Mineralogy and Petrology 113:524–532.

[maps13494-bib-0043] Xu C. , Zhao S. , Li C. , and He X. 2016 Plagioclase twins in a basalt: An electron backscatter diffraction study. Journal of Applied Crystallography 49:2145–2154.

